# Market orientation and technological orientation in business services: The moderating role of organizational culture and human resources on performance

**DOI:** 10.1371/journal.pone.0270737

**Published:** 2022-06-29

**Authors:** Krzysztof Borodako, Jadwiga Berbeka, Michał Rudnicki, Mariusz Łapczyński, Mariusz Kuziak, Krzysztof Kapera

**Affiliations:** Institute of Management, Cracow University of Economics, Kraków, Poland; Ghazi University, PAKISTAN

## Abstract

Contemporary conditions of the functioning of enterprises mean that they are increasingly looking for opportunities to improve organizational performance in strategic management. Scientists are looking for optimal solutions, an appropriate combination of assets and resources, so the debate in the field of strategic orientations is still valid and gaining in importance. Several studies have explored the construct of market orientation, but few include technological orientation with the moderating effects of company assets. In the era of the highly competitive technology market, the area of technological business service providers are particularly interesting, but still undiscovered. This paper examines the effects of market orientation and technological orientation on organizational performance with the inclusion of organizational culture and human resources as moderators. Using questionnaire responses from technological business service providers (n = 689), a regression analysis was conducted to confirm the hypotheses. The results established evidence of positive relationships between market orientation—organizational performance and technological orientation—organizational performance, although in technological firms, the market orientation had a stronger correlation with organizational performance than the technological orientation. Moreover, the organizational culture and human resources play a moderating role in the relationships of market orientation—organizational performance and technological orientation—organizational performance, while weak human resources management weakens relationships market orientation—organizational performance and technological orientation—organizational performance and strong organizational culture reduce the effect of market orientation on organizational performance, significantly reducing the effect of technological orientation on firm performance.

## Introduction

Interest in strategic orientation and its impact on organizational performance is constantly growing. Two types of strategic orientation seem to be interesting in the context of current scientific debate [1–3]. First is a market orientation that has been intensively researched from the 1990s [4, 5] to the present. Most recognized definitions of market orientation present it as “*a culture in which all employees are committed to the continuous creation of superior value for customers*” [6, p. 242]. This concept focus not only on existing customers but also include potential customers and competitors [7]. It is commonly accepted that market orientation has a positive effect on performance. The second concept is technological orientation [8, 9], understood mostly as technological knowledge and innovation. Technological orientation can be defined as the extent to which companies place emphasis on obtaining and making use of advanced technologies in developing new and existing products [10]. It is widely acknowledged that technology makes vital contributions to stimulate company processes and optimize resource usage. Nowadays, highly dynamic technological change plays an important role in market competition, innovation development, and successful business running. In this study, these two orientations (market and technological) were evaluated and compared from the standpoint of the impact on firm performance.

In company management, assets such as organizational culture and human resources—perceived as human capital, are paramount [11, 12]. Their impact on firm performance has already been confirmed, but they have been sporadically studied in the context of services [13]. It is true that organizational culture and human resources were also used in management and marketing literature as moderators [14, 15], but they have not been used so far in relation to various types of strategic orientation. This relation is of particular interest because strategic orientation (in this case market and technological) could be seen as a key element of organizational culture [16]. Additionally, many scholars stress the importance of human resource management, creativity, and knowledge of employees in the context of the strategic orientation [17, 18].

Although we know a lot about market orientation, its relationship with technological orientation is still insufficiently researched—in particular in the technological business services (TBS) sector. This should be considered as an area requiring further exploration in the era of the highly competitive technology market. However, when looking for factors to build this competitive advantage, organizational culture and human resources can be considered as crucial. Their special role, as it is understood according to the resourced-based theory, results from the relatively high level of difficulty competitors face in imitating these resources.

We still do not know much how human resources and organizational culture of the enterprise moderate the impact of market orientation and technological orientation on the organizational performance of companies (in particular technological service companies). It is also worth knowing how these two elements interact in isolation, but also in mutual interaction on performance.

The goal of this research is to study the possible effect of organizational culture and human resources (separately and simultaneously) on the relationships between market orientation and organizational performance, but also between technological orientation and organizational performance. This research contributes to the knowledge of strategic (market and technology) orientations in five ways. First, it confirms the positive effect of market orientation on business performance (and for a specific group of technological business services). Second, this study addresses the knowledge gap associated with the relationship between technological orientation and business performance in the business services sector. The third area of contribution concerns specific moderators (organizational culture and human resources) that influence the relationships of the studied strategic orientations (market and technological orientations) on business performance. The fourth contribution to the marketing and management literature is to offer an integrated model that includes elements of the company’s resources (organizational culture and human resources), forms of strategic orientation (market and technological orientations), and business performance. The fifth area to which this research of contribution of this research is providing evidence of the critical role of strategic management concepts in service enterprises related to technology as a core activity area.

## Literature review

### Market orientation

Market orientation describes the company’s attitude towards understanding its customers and meeting their needs [4]. It takes into account both, current needs (responsive market orientation) and future or latent needs (proactive market orientation) [19, 20].

Concerning behavioral approaches, Kohli and Jaworski [21] argue that market orientation reflects how companies manage and reply to the gathering and dissemination of market data throughout useful areas, and proper response to accumulated intelligence. One of the tools used by companies for increasing value for customers is the implementation of marketing orientation that is based on the creation of desired organizational behavior leading to better performance. The cultural perspective concerning market orientation emphasizes the importance of both customers and competitors as strategic means of identifying customer’s needs. In accordance with the principles of organizational culture cooperation between different teams in the company ensures a deeper understanding of customer needs and a higher level of customer satisfaction [4, 22].

Market orientation is among best-studied concepts in strategic marketing literature, and its tremendous effect on performance is broadly recognised [23–25]. Researchers have suggested market orientation as a key performance enhancer hence more success [26]. Along with the development of basic research perspectives, the basic scales of business performance measurement (MKTOR and MARKOR) were developed and refined. At the same time, along with the development of technology, the need to expand and supplement the research on market orientation was noticed. This assimilation of IT and marketing gave rise to a new research area, commonly known as e-marketing, integrating market orientation, technologies and their impact on company performance [27–30].

Despite much evidence of the positive impact of market orientation on performance, there has been little research done in the context of business services [28]. In order to fill this gap, the impact of market orientation on performance in business services perspective will be examined first. Therefore, we propose:

***Hypothesis 1: Market orientation has a positive effect on organizational performance***.

### Technological orientation

Another approach which is often compiled with market orientation is technological orientation. It is a managerial technique that emphasises using technology in all dimensions of the company’s operations concerning both products and procedures [2, 31]. Technological orientation describes the company’s attitude towards engaging in research, technological development, analysing technological potential, and forecasting technological trends [32]. Technological orientation can be defined as the extent to which companies place an emphasis on obtaining and making use of advanced technologies in developing new products and developing those already existing. Often, this is connected to corporate activities that inspire openness to fresh ideas, innovative thinking and proactive initiation of important decisions [10].

Referring to the resource-based view [33], technological resources are a key source of company innovation and the development of basic competencies [34]. Therefore, technological orientation can be considered crucial in a company’s success [35, 36].

Research concerning the influence of technological orientation on performance often connects technological orientation and market orientation, showing strong interdependencies [34, 37–39]. Studies on strategic orientations have recognized numerous methods wherein multiple orientations can be followed by the same company to fulfill the evolving desires of the business context or to help new and changing targets for the company [40, 41].

Empirical studies in large part underline a positive association between technological orientation and business performance. Such research additionally advise that a technological orientation may also have a more beneficial effect on performance with increasing degrees of turbulence regarding each markets and technologies [32, 42]. The feature that characterizes business services firms is knowledge [43, 44], and one of its main components are technological capabilities [37, 45]. Therefore it is surprising that little empirical studies has been carried out yet to take a look at the connection between a technological orientation and business services performance. Therefore, we propose:

***Hypothesis 2: Technological orientation has a positive effect on organizational performance***.

### Organizational culture

Schein [46] defines organisational culture as a norm of common primary assumptions developed by a group. Organisational culture is developed through shared experiences of success in solving problems. It is so successful that it becomes worthy of teaching new employees as the proper way to perceive, think, and react in relation to those issues.

Narver & Slater [4] claim that organisational culture is the main element of market orientation introducing the necessary attitudes in the company leading to offering customers products that meet their expectations and thus generating better results for the company. So organisational culture is embedded in market orientation and therefore it can be expected significant relations exist between them.

The influence of organisational culture on a company performance is widely discussed in the literature [47]. Empirical findings are provided, among others, for small and medium enterprises [48] in financial services [49]. According to De Long [50], organisational culture is one of the three main factors influencing any knowledge management (KM) strategy designed to improve business performance.

Hartnell, Ou, Kinicki, Choi, & Karam [51] stress that organisational culture is an important predictor of organisational effectiveness. A supportive organisational culture encourages employees to acquire, generate and transfer knowledge in the most effective way [52].

Market orientation requires customer oriented culture. In service providing companies one of the crucial issue of creating relations with clients is high quality of services [53]. Supportive organisational culture directly impacts the organisational performance and moreover influences other elements of the strategy. Based on the above premises, the next hypothesis is proposed:

***Hypothesis 3:Organisational culture has a moderating effect on market orientation and organisational performance relationship***.

The definition of technological orientation by Deshpande, Grinstein, Kim, & Ofek [10] clearly stresses that it requires openness to new concepts, creative approach, and proactive actions. These elements are significantly shaped by organisational culture. Especially the adhocracy culture supports values like: creativity, entrepreneurship and risk-taking [54]. Assuming that organisational culture can have impact on technological orientation and performance relationship we formulate the following:

***Hypothesis 4: Organisational culture has a moderating effect on technological orientation and organisational performance relationship***.

### Human resources

Human resources consists of employees who are one of the basic pillars of the organization. Gaining a competitive advantage and sustaining it depends on their performance. Employees are involved in the process of achieving a group or individual goals [55].

Human resources create one of the most important company assets–human capital. According to Curado & Bontis [56] human capital is equated with employees and covers not only the competencies and knowledge of employees but also their skills in connection with their commitment and professional experience. A firm’s performance relies on the quality of its human resources [57] and job satisfaction [36]. Schiuma & Lerro [58] stress the importance of ensuring an appropriate balance of educational profiles within the organization. Richard [59] further highlights the need for a diverse stock of human capital. An interesting research issue is the recognition of the role of human resources as a moderator of the influence of a given orientation on the company performance.

As it was stressed above, the pillars of market orientation are: consumer-centred view, intelligence generation and dissemination, responsiveness [60]. In all these issues, employees are crucial for efficient task realisation [61]. That’s why the variable human resources can be expected to be a moderator in market orientation and organizational performance relation. Taking above arguments under consideration the another hypothesis is proposed:

***Hypothesis 5: Human resources have a moderating effect on market orientation and organizational performance relationship***.

In companies with technological orientation, technologies act as a means of transferring, disseminating and adapting knowledge to the specific operating conditions of companies providing business services [62]. Knowledge is embedded in human resources and moreover, human resources absorb knowledge to use it effectively resulting in better performance [63]. So it can be expected that human resources have a significant relation with the organizational performance. That’s why the variable human resources can be taken as a moderator in the technological orientation and organizational performance relation.

***Hypothesis 6: Human resources have a moderating effect on technological orientation and organizational performance relationship***.

Human resources and organisational culture are closely related in a company and many concepts explain these relationships. In the field of human resources, the leader and his influence on organisational culture are often paramount distinguished [64]. The model proposed by Yuan & Lee [65] links together the concepts of leadership performance, of organizational culture and of employees’ performance within a system of multiple influences. The direct influence of the leader is claimed by Mumford, Connelly & Gaddis [66]. The indirect influence is highlighted where leaders impact employees attitude and motivation by influencing the nature of both the work environment and organizational culture [64]. Thus, a synergy effect of human resources and organisational culture interaction occurs and impacts performance of companies with both market orientation and technological orientation. Based on these arguments two hypotheses are formulated:

***Hypothesis 7: Organisational culture and human resources simultaneously have a moderating effect on market orientation and organisational performance relationship***.

***Hypothesis 8: Organisational culture and human resources simultaneously have a moderating effect on technological orientation and organisational performance relationship***.

### Research model

**Fig 1:**
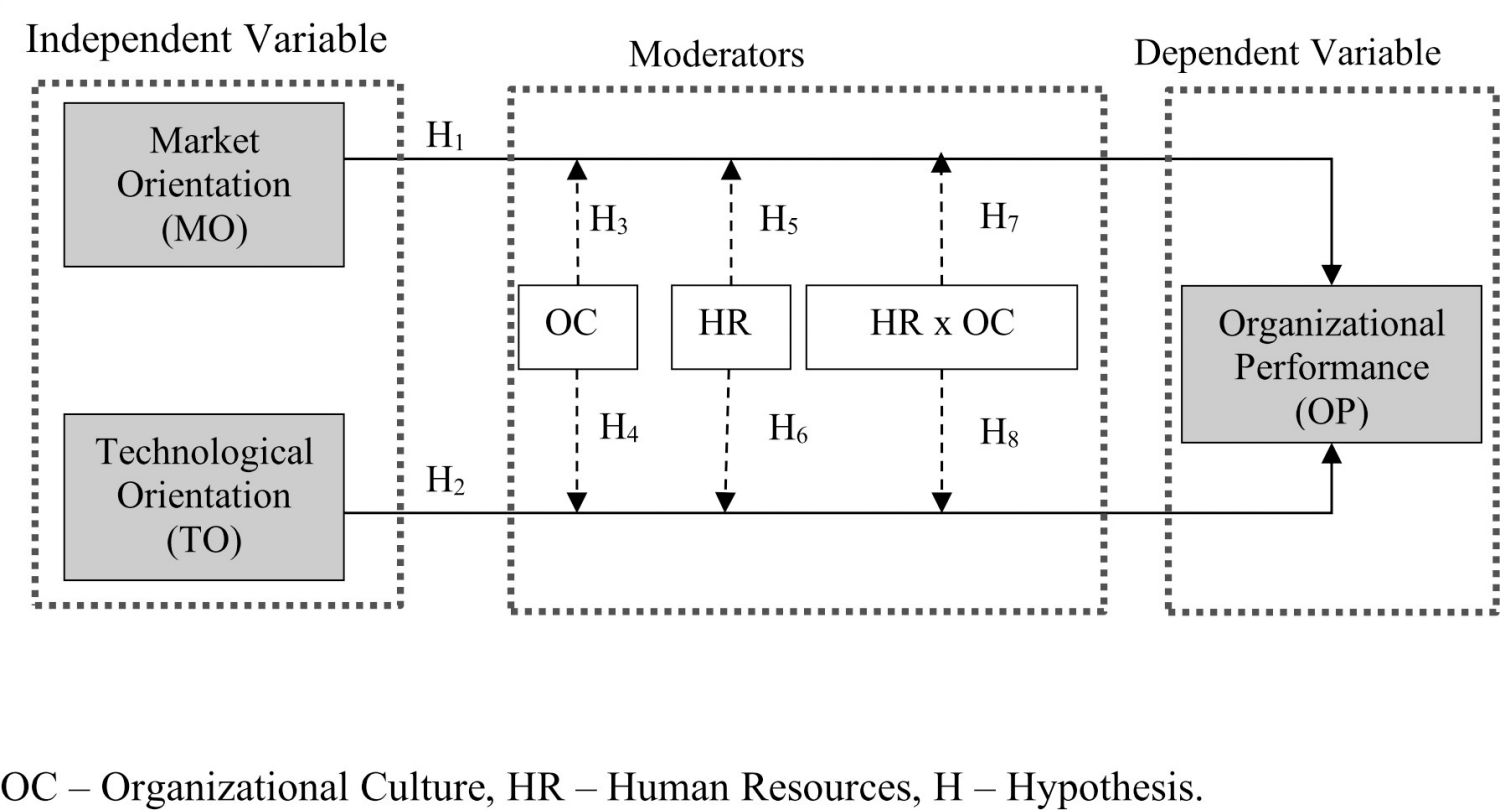
The proposed research model.

In this study, hypothesized variables, moderators, and their relationships resulted from a management and marketing literature review establishing the following research model (Fig 1). Grounded in the resource-based view approach, a model was developed assuming the impact of strategic orientation (market and technological) on the company’s performance with the moderators on these associations being organizational culture, human resources, and their combined effect.

The model–multivariable linear regression equation–can be expressed as

$$OP=\beta_{0}+\beta_{1}MO+\beta_{2}TO+\varepsilon_{i}$$

where OP (organizational performance) is dependent variable, MO (market orientation), TO (technological orientation) are independent variables, β_0_ is intercept, β_1_ and β_2_ are parameters and ε_i_ is residual value. The analysis was carried out in subsets of the data set determined with the use of moderating variables HR and OC.

## Methodology

### Ethics clearance, sample and data collection

The research was conducted as a grant project financed by Polish National Science Center, which approved during the project evaluation any ethical requirements.

This research involves the processing of personal data (name of the respondents and the email) used in the questionnaires, which was handled in with the accordance of EU General Data Protection Regulation (GDPR). Personal information of the respondents and their responses have been kept and analyzed anonymous.

In our study, we secured the confidentiality, anonymity, and informed consent of participants (the participant agreed to take a survey after the information what will happen to the answers they provide—referring to the company activities). Informed consent of the participants was assumed upon the return of the completed survey.

Participation in the survey was 100% voluntary, and a participant could choose whether to begin the survey or if they’ve already begun it, complete the survey. Our study was conducted in frames of social science and it is related to the firm activities. The informants are humans, but the information collected from them refers to their company (not themselves).

The data collection process was conducted with Lime Survey, an open-source software hosted on the university server. The respondents were contacted by e-mail. The initial sample of respondents was acquired from a database derivative of the official court registry of business activity. The final mailing list consisted of 50,023 e-mail addresses. The data collection process was started on June 9, 2019, by systematic sending out invitations (for random company samples) with a unique URL to the survey. A follow-up mailing was sent a month later to the respondents who started completing the questionnaire but had not finished the process and to those who had not reacted to the first mailing at all. Additional reminders were sent to the selected respondents at the turn of September and October 2019. The data collection process resulted in 689 fully completed questionnaires, whose dominant areas of activity are IT services (42.5%), technical research and analysis services (10.6%), and engineering and architecture services (46.9%). The sample description is presented in Table 1.

**Table 1:** Sample description.

Market presence	Items	%	Size [employees]	Items	%	Industry	Items	%
**Up to 2years**	64	9.29	**Up to 9**	523	75.91	**Technical research and analysis**	73	10.60
**2-5y**	138	20.03	**10–49**	102	14.80	**IT services**	293	42.52
**6-10y**	171	24.82	**50–249**	41	5.95	**Engineering and architecture services**	323	46.88
**11-20y**	156	22.64	**Over 249**	23	3.34			
**Over 20y**	160	23.22						

### Measures

#### Organizational performance–dependent variable

To be consistent with previous research of strategic orientation, we incorporated three measures of business performance: sales growth, client satisfaction and successful service launch. The respondents were asked to assess their business performance in the last three years, in order to reduce any biases related the dynamic changes in one particular year. The sales growth reflects the financial performance of the firm, while client satisfaction was related to the quality of the service and the communication with clients. All these indicators were judgmental measures [42, 67], commonly applied in the strategic orientation research.

#### Independent variables

The explanatory variables in this study are the two orientations defined and described in the previous sections: technological and market orientation. *Market orientation* and T*echnological orientation* scales consist of four items (each rated on a 5-point Likert scale), which help assess the validity and reliability of the construct.

#### Moderator variables

A moderator variable in a regression model is an additional variable that can change the relationship between the independent variables X and the dependent variable Y (size or sign of the effect of X on Y) [68]. Moderation (interaction) can be studied by introducing qualitative variables (e.g. gender) or quantitative variables (e.g. age). Due to the fact that two dichotomous variables were used during the study: organizational culture and human resources, a subgroup analysis was conducted to detect moderators. Each moderator variable was measured on a 5-point Likert scale adapted from the strategic orientation literature [69, 70]. We split moderator variables at the median value to build the high and low subgroup for each moderator and create four subgroups of the sample with high or low organizational culture and human resources.

To examine the above-proposed hypotheses we employed multiple regression analyses as previously applied by Cheng [71] and Ho, Plewa, & Lu [72].

### Method

Multiple regression investigates the effect of many independent variables (X) on one dependent variable (Y.) The general multiple regression equation can be represented by the formula

$$y=b_{0}+b_{1}x_{1}+b_{2}x_{2}+\cdots +b_{n}x_{n}+\varepsilon$$

where b_1_, b_2_, … b_n_ are coefficients which allow to explain or predict the dependent variable Y. This is a very popular analytical tool widely used in many areas [e.g. 73–76]. For the purposes of this analysis, the co-linearity of the variables was diagnosed using the tolerance coefficient. Moreover, the assumptions of homoscedasticity and normal distribution of residuals were checked.

## Results and analysis

### Reliability, validity and descriptive statistics

As defined by Ginty [77], construct validity is “the extent to which the measurements used, often questionnaires, actually test the hypothesis or theory they are measuring”. Two types of construct validity–we assessed (convergent and discriminant validity) as part of the analyses. The approaches were based on Cronbach’s alpha, composite reliability, average variance extracted (AVE), and cross-loading values [78]. Table 2 presents the descriptive statistics and correlations of the observed variables.

**Table 2:** Basic descriptive statistics of the constructs.

Variables		Mean	Standard deviation	1	2	3	4	5	6	7	8	9	10	11
1	TO1	4.04	1.05											
2	TO2	3.85	1.13	0.76										
3	TO3	3.72	1.14	0.63	0.70									
4	TO4	3.6	1.26	0.51	0.57	0.66								
5	MO1	3.8	1.16	0.52	0.57	0.51	0.48							
6	MO2	3.65	1.15	0.53	0.61	0.60	0.54	0.75						
7	MO3	3.82	1.11	0.53	0.58	0.55	0.50	0.69	0.82					
8	MO4	3.8	1.11	0.55	0.62	0.58	0.53	0.69	0.82	0.87				
9	P1	3.27	1.03	0.42	0.51	0.51	0.49	0.53	0.60	0.54	0.58			
10	P2	3.27	1.18	0.33	0.34	0.34	0.31	0.36	0.36	0.32	0.37	0.50		
11	P3	3.85	0.98	0.49	0.46	0.42	0.37	0.47	0.48	0.50	0.52	0.50	0.53	

Sample size = 689, p<0.001

TO–technological orientation, MO–market orientation, OP–organizational performance

In the first step of the analysis, confirmatory factor analysis was used to investigate construct validity. Values of RMSEA = 0.065, chi2 (df) = 159.852 (41) p <0.001, McDonald’s NCI = 0.917, AGFI = 0.854 indicate an acceptable fit. Table 3 shows Cronbach’s alpha and composite reliability results. The former range from 0.753 to 0.932, while the latter exceed the threshold value of 0.7. This is a satisfactory result, which proves the good reliability of the measurement model. The last column of the table contains AVE values. Since they are all higher than the cut-off value of 0.5, the convergent validity is acceptable [79].

**Table 3:** Results of confirmatory factor analysis.

Construct	Items	Cronbach’s Alpha	Composite Reliability (CR)	Average Variance Extracted (AVE)
TO	4	0.874	0.937	0.789
MO	4	0.932	0.979	0.920
OP	3	0.753	0.807	0.582

TO–technological orientation, MO–market orientation, OP–organizational performance

The correlation coefficients between the constructs (TO, MO and OP), and on the diagonal the square roots of the AVEs were presented in Table 4. According to the Fornell & Larcker [79] criteria, they are higher than the correlation coefficients, which proves a satisfactory discriminant validity.

**Table 4:** Result of correlations and square root of AVEs (on the diagonal).

	Mean	Standard deviation	TO	MO	OP
TO	15.213	3.907	0.888		
MO	15.064	4.124	0.705	0.959	
OP	10.395	2.615	0.586	0.620	0.762

TO–technological orientation, MO–market orientation, OP–organizational performance, p < 0.001

To establish discriminant validity cross-loadings were also assessed (Table 5). The factor loadings for observed variables are highest for the construct with which they are associated. This confirms the previous conclusion that the measurement model has good discriminant validity.

**Table 5:** Results of cross-loadings.

	TO	MO	OP
TO-1	**0.786**	0.257	0.225
TO-2	**0.794**	0.352	0.202
TO-3	**0.796**	0.312	0.193
TO-4	**0.715**	0.297	0.177
MO-1	0.316	**0.746**	0.245
MO-2	0.356	**0.831**	0.218
MO-3	0.306	**0.864**	0.182
MO-4	0.345	**0.828**	0.242
OP-1	0.310	0.455	**0.565**
OP-2	0.145	0.104	**0.890**
OP-3	0.260	0.316	**0.709**

TO—technological orientation; MO—market orientation; OP–organizational performance

### Hypotheses testing—Results

Multiple regression analysis was carried out to test the proposed hypotheses (Table 6). In companies providing technological services, there is a significant relationship between the orientation of companies and their organizational performance. In particular market orientation and technological orientation are related to their organizational performance (see Table 6). The positive and significant coefficients (β = 0.295 for technological orientation and β = 0.412 for market orientation, p = 0.000)—provide strong support for H_1_ and H_2_. The strength of the relationship is significantly higher in the case of market orientation. This shows that even in technological companies, the determination to possess and implement new technologies is not enough to achieve high organizational performance. The results indicate that market orientation, focused on clients and creating (co-creating) value for them is more effective.

**Table 6:** Multiple regression results.

Moderators	MO				TO				R2
	Coefficient (95% confidence interval)	SE	t	p	Coefficient (95% confidence interval)	SE	t	p	
Technological business service providers	0.412 (0.331; 0.492)	0.041	10.131	0.000	0.295 (0.214; 0,376)	0.041	7.238	0.000	0.428
Low level of OC	0.336 (0.228; 0.444)	0.056	6.044	0.000	0.344 (0,236; 0,452)	0.056	6.174	0.000	0.389
High level of OC	0.383 (0.275; 0.491)	0.055	7.004	0.000	0.131 (0,023;0,239)	0.055	2.388	0.018	0.204
Low level of HR	0.349 (0.225; 0.473)	0.063	5.577	0.000	0.262 (0,138; 0,386)	0.063	4.195	0.000	0.314
High level of HR	0.429 (0.332; 0.525)	0.049	8.731	0.000	0.218 (0,122; 0,314)	0.049	4.434	0.000	0.321
Low HR + Low OC	0.329 (0.315; 0.343)	0.007	4.749	0.000	0.319 (0,305; 0,333)	0.007	4.608	0.000	0.354
Low HR + High OC	0.118 (-0.137; 0.373)	0.128	0.920	0.361	-0.026 (-0,281; 0,229)	0.128	-0.206	0.837	0.013
High HR + Low OC	0.281 (0.109; 0.453)	0.087	3.238	0.002	0.321 (0,149; 0,493)	0.087	3.694	0.000	0.245
High HR + High OC	0.465 (0.349; 0.581)	0.059	7.863	0.000	0.103 (-0,013; 0,219)	0.059	1.745	0.082	0.262

Remark: Estimates (coefficients) refer to the relationship between the independent variables MO and TO and the dependent variable OP.

#### Moderating effect of organizational culture

The results on the importance of organizational culture as a moderator of the relationship between companies’ orientation and their organizational performance are summarized in Table 6. The division of the role assigned to organizational culture was taken into account, differentiating it into high and low levels. In companies providing technological services, the low level of organizational culture has a moderating effect on the dependence of the orientation of companies and their organizational performance—similar to the strength in the case of both orientations. In companies characterized by market orientation, the moderating effect of low level of organizational culture consists in reducing this dependence. It is significant (0.336) and it supports H_3_. On the other hand, in companies with technological orientation, low level of organizational culture increases the dependence of orientation and performance. The moderating effect of low level of organizational culture (0.344) on technological orientation and organizational performance supports H_4_. The high level of organizational culture has a moderating effect on the relationship between both technological orientation and organizational performance (0.131) as well as market orientation and the organizational performance (0.383) which supports H_3_ and H_4_ respectively. In companies with both orientations the high level of organizational culture reduces the relationship. However, it should be noted that the moderation effect of high organizational culture is almost three times stronger for market orientation than for technological orientation.

#### Moderating effect of human resources

Human resources assets have a moderating effect on the impact of market orientation on the company’s performance and technological orientation of a company on its organizational performance. Taking into account the importance attached to human resources in companies, high and low level were distinguished and examined. The results (see Table 6) prove that disregarding the importance attached to human resources (low HR) weakens the relationship between both market orientation and organizational performance (0.349) and technological orientation and organizational performance (0.262), although both results are significant what provides support for H_5_ and H_6_. The high importance attached to human resources in companies (high level of human resources) had a moderating effect on the market orientation—organizational performance relationship (0.429), namely strengthening it. In the technological business service providers with technological orientation, high levels of human resources had a moderating effect on technological orientation—organizational performance relationship (0.218), although it weakens the relationship between orientation and performance. Supporting human resource development in the case of market orientation allows for a stronger influence on organizational performance.

#### Moderating effect of human resources and organizational culture

The next part of the research was the identification of moderation effects of human resources and organizational culture, taking into account simultaneously the different levels (high and low) of both independent variables. Thus 4 variants were taken into account–see Table 6. The statistically significant values of the regression equation obtained support: H_7_ and H_8_. When examining the simultaneous impact of a low level of human resources and a low level of organizational culture, a moderating effect occurs, with a similar strength of impact in both technological and market-oriented companies. The moderating effect also occurs for companies with a high level of human resources and low organizational culture, for companies with both market orientation (0.281) and technological orientation (0.321). This effect is stronger in the case of companies with technological orientation than market orientation. In companies providing technological services, the relationship between the combination of a high level of human resources and a high level of organizational culture (both moderators simultaneously) and connection technological orientation—organizational performance was negative but not significant (p>0.05). On the other hand, a high level of human resources and a high level of organizational culture increases the moderation effect on the relation market orientation—organizational performance. Referring to effect sizes of relevant estimated coefficients in high values of both moderators (high HR and high OC) we observed four times higher values of coefficients for market orientation (0.465) than for technological orientation (0.103), while for both low values of moderators (low HR and low OC) these coefficients were quite similar (market orientation– 0.329, technological orientation– 0.319).

## Discussion and theoretical contribution

The stated goal of this research is to examine the effects of MO and TO on organizational performance through the incorporation of organisational culture and human resources as the moderators. This study contributed to the current literature in five ways.

**First**, in a specific sample of technological BS, this study confirms as stated in the previous literature [40, 80, 81] the positive affect of MO on business performance. An interesting element of our study refers to higher importance of a relationship between MO and performance than TO related to performance, although the research sample relates strictly to technological BS. The MO comprise customers’ needs recognition, unique and high value added by the delivered services, and problem solutions offered by products for customers. All these aspects are closely connected with innovation activities which definitely affect firm performance and have been the subject of numerous studies [80, 82–84]. This study confirms the importance of MO in organizational performance even in companies delivering services closely connected with technology.

**Second**, this research fills an important research gap by examining the relations of TO with business performance in the service industry. Aspects of TO in this study included easy acceptance of new technologies by the company management, systematic scanning for new technologies, usage of top technologies for the industry, and usage of ICT (Information and Communication Technology) as a source of competitive advantage. The concept of TO is strictly connected with technological expertise which can be a key factor in generating a competitive advantage by successful knowledge management [36, 61, 85]. Measures adapted in this study cover the critical aspect of service development and firm progress. From the theoretical point of view, it could be very important that the firms from the sample composed of technological businesses still underestimate the role of technology (related to TO) when compared to MO. Nevertheless, the empirical verification of this research confirms the statistical significance of TO in successful organizational development.

**The third** contribution to the knowledge base refers to specific moderators (OC or HR) of these both relationships: MO-OP, TO-OP and MO-OC-OP, MO-HR-OP, TO-OC-OP, TO-HR-OP analysed separately. The results of this study confirm that the moderating role of **OC** in relation to MO-OP as well as to TO-OP are statistically significant. These results support previous studies related to the MO and OC [86, 87]. The results confirm that low level of OC could risk undermining the impact of MO on OP. The results indicate that inclusion of an OC moderator into the model decreases the relationship between the MO and OP (0,336 and 0,383 vs 0,412). Obtained empirical results could be seen in line with Zhou, Li, Zhou, & Su [35] who revealed that fostering MO culture in the company does not necessarily start better OP. This culture should be implemented within the whole organization; generating proper employee’s behaviors considering internal and external technological conditions. We included into the model human resources (HR) as an important asset determining the strategic orientation of the firm. The knowledge and skills offered by the **HR** can influence the relation between the MO and organizational performance. Thoughtful efforts to develop HR lead to successful processing of task implementation and impacts positively on the quality of customer service. This examination reveals that lower attention put on HR leads to weaker relationships between MO and performance, but also weaker TO-performance relationships. Opposite tendencies appear in cases of firms paying high attention to HR. In those firms (with high HR), the MO-OP relationship increased (over the level of model without the moderators 0,429 vs 0,412), but it should be stressed that there is very strong decrease of the significance of technology. In these circumstances, investment in human capital leads to the decrease of TO-performance relationships.

**The fourth**, this research contributes to the marketing and management literature by integrating the MO, TO, OC, HR and organizational performance in a single research model. This investigation may be among the first to theoretically argue and empirically verify the multifaced relationships among the above mentioned constructs. These relations could be presented as two paths MO-(HRxOC)-OP and TO-(HRxOC)-OP. Formerly there have been no empirical studies implementing all these constructs in the single model while highlighting the crucial background of research which is business services. The findings reveal interesting patterns among these variables. The companies with low level of HR and OC tend to reduce the significance of the MO-performance relationship, but slightly increase the role of technology in generating better performance. This empirical study justifies the importance of OC and HR management in business development. Otherwise these two areas of the company contribute to reducing the impact of marketing activities and creating a place for competitors in the market. Such a strategy would be counterbalanced by the greater importance of technology in creating a competitive advantage—what also stressed Limbu et al. [36], but does not ensure as good a position as in the case of the high role of OC and HR.

**Finally** this study provide evidence of the significance of some strategic management concepts (MO, TO, OC, HR) in the field of business services. As mentioned above this finding reinforces the opinion that technological orientation and market orientation should be treated as drivers for competitive advantage based on original and difficult to copy organizational culture and human resources integrated within strategic management processes.

## Managerial implications

From a practical standpoint, the findings deliver guidance to managers of technological service businesses on how to achieve superior performance. The results of examination suggest that both orientations (market and technological) can lead business services firms to better performance. However, introduction of market orientation by the company would give it better performance than focusing on developing new technologies to achieve technological leadership. Managers should also understand that the higher or lower importance of organizational culture or human resources can significantly influence the market position. Top managers should pay close attention to organizational culture and human resources management, simultaneously monitor the knowledge development of the employees while taking care of their active knowledge acquirement and motivating them to invent new services. All these activities should be run with active participation of employees on the whole service process. Managers should understand the high role of appreciation awards for new technological ideas with commercialization possibility.

Besides managerial implications, we would like to draw attention to the impact of our research in the social and environmental areas. The social aspect of this research refers to the discussion regarding technology as a critical factor determining high economic development of societies. Our research shows that, on the one hand, the human factor (human resources) and the contextual factor (organizational culture) have a large impact on the performance of market- and technology-oriented companies. On the other hand, even technology-dominated companies with a prime market orientation must not forget the role of employees in shaping an atmosphere conducive to creativity and openness to new challenges. In the environmental field, this research can inspire decision-makers responsible for the sustainable development of regions and countries in which technology-based business service companies operate. Supporting technologically oriented and innovative companies can lead to the further development of eco-innovation and thus reduce the negative impact of human activities on the environment. As our research indirectly shows, regions and countries should support such firms to increase the environmental knowledge of their employees and build environmental awareness in the organizational cultures of these companies.

## Limitations and future research

In this study we identified several limitations that could be included in forthcoming research. First, we used a measure of business performance based on subjective assessment of respondents included sales growth, clients’ satisfaction and successful service launch. As we mentioned, this methodology is commonly adapted in the strategic orientation research but in the future, it would be worth taking into account secondary data of the surveyed companies. This would undoubtedly verify the respondents’ assessments, giving the results greater objectivity. As for data, our research is based on questionnaire, which narrows the possibility of in-depth analysis of the terms of identifying the leading orientation characterizing a given company as well as decisions regarding marketing management. Future research based on qualitative research methods may provide additional information on the different strategies of action by giving a clearer picture of the orientations identified. Secondly, our research adopted moderators (human resources and organizational culture), which are strongly related to national culture, mentality, etc., which strongly determine the patterns of decisions made. Therefore, in the future, it would be necessary to take into account the need to conduct these studies, where the research sample would be companies with their headquarters in different countries.

Our study also represents a snapshot for a particular period and present only a fragment of business services (IT, Technical research and analysis, Architectural and engineering services). Therefore, there is a need to repeat these studies in the future, including other industries classified as business services. Studies of other business services industries, due to the different specificity of the business, may show some differentiation in the scope of the impact of human resources and organizational culture on market orientation and technological orientation in achieving better organizational performance.

## Supporting information

S1 Appendix (PDF)

S2 Appendix (PDF)
