# Supplementary material for: Market orientation and technological orientation in business services: The moderating role of organizational culture and human resources on performance
Source: PLoS One. 2022 Jun 29;17(6):e0270737. doi: 10.1371/journal.pone.0270737 (PMC9242498; doi:10.1371/journal.pone.0270737)
Supplement: S1 Appendix — (PDF) [file pone.0270737.s001.pdf]

Questionnaire form

The following rating scale description was used throughout the study: (1) Strongly disagree; (2) Disagree; (3) Neither agree nor disagree; (4) Agree; (5) Strongly agree.

Code	Statements	Rating
Organizational culture (OC)		
OC1	*Senior management actively seeks innovative ideas.*	1 – 2 – 3 – 4 – 5
OC2	*In our organization, we discuss new ways of doing things.*	1 – 2 – 3 – 4 – 5
OC3	*Initiatives often receive a favorable response in our company, so people feel encouraged to generate new ideas.*	1 – 2 – 3 – 4 – 5
OC4	*Employees that resist innovation or perceive innovation as too risky are the exception rather than the rule.*	1 – 2 – 3 – 4 – 5
Human resources (HR)		
HR1	*We expect high innovativeness and creativity from our employees.*	1 – 2 – 3 – 4 – 5
HR2	*Training programs to increase personal innovativeness are offered regularly.*	1 – 2 – 3 – 4 – 5
HR3	*The performance of employees regarding innovativeness is systematically assessed and evaluated.*	1 – 2 – 3 – 4 – 5
HR4	*Our employees are encouraged to actively seek innovative ideas.*	1 – 2 – 3 – 4 – 5

Code	Statements	Rating
Market orientation (MO)		
MO1	*We continuously try to discover additional needs of our customers of which they are unaware.*	1 – 2 – 3 – 4 – 5
MO2	*The newly developed services of our company offer unique advantages to our customers.*	1 – 2 – 3 – 4 – 5
MO3	*The newly developed services of our company solve the problems of our customers.*	1 – 2 – 3 – 4 – 5
MO4	*The newly developed services of our company deliver high benefits for our customers.*	1 – 2 – 3 – 4 – 5
Technological orientation (TO)		
TO1	*New technological innovations are readily accepted and used in our firm.*	1 – 2 – 3 – 4 – 5
TO2	*We systematically scan for new technologies inside and outside the industry.*	1 – 2 – 3 – 4 – 5
TO3	*We use technologies that have an impact on or cause significant changes throughout the industry.*	1 – 2 – 3 – 4 – 5
TE4	*Information systems/information technology is a source of competitive advantage in our firm.*	1 – 2 – 3 – 4 – 5

Code	Statements	Rating
Organizational Performance (OP)		
P1	*Our business has a special ability to launch successful new services.*	1 – 2 – 3 – 4 – 5
P2	*The growth of sales in our company can be described as successful.*	1 – 2 – 3 – 4 – 5
P3	*Customer satisfaction with our company can be described as successful.*	1 – 2 – 3 – 4 – 5

Information about the respondents

Professional experience:	Business role:
○ less than 2 years<br>○ 2-5 years<br>○ 6-10 years<br>○ 11-20 years<br>○ over 20 years	○ CEO<br>○ Director<br>○ Manager<br>○ Specialist

Information about the respondents' companies

Market presence:	Size (number of employees):	Type of services
○ Up to 2 yrs.<br>○ 2-5 yrs.<br>○ 6-10 yrs.<br>○ 11-20 yrs.<br>○ Over 20 yrs.	○ Up to 9 employees<br>○ 10-49<br>○ 50-249<br>○ Over 249	○ IT services<br>○ Technical research and analysis services<br>○ Engineering and architectural services
