# Supplementary material for: Market orientation and technological orientation in business services: The moderating role of organizational culture and human resources on performance
Source: PLoS One. 2022 Jun 29;17(6):e0270737. doi: 10.1371/journal.pone.0270737 (PMC9242498; doi:10.1371/journal.pone.0270737)
Supplement: S2 Appendix — (PDF) [file pone.0270737.s002.pdf]

Formularz ankiety

W całym badaniu zastosowano następujący opis skali ocen: (1) Zdecydowanie się nie zgadzam; (2) Nie zgadzam się; (3) Ani zgadzam się, ani nie zgadzam; (4) Zgadzam się; (5) Zdecydowanie się zgadzam.

Kod	Stwierdzenia	Ocena
Kultura Organizacyjna (OC)		
OC1	*Kierownictwo firmy aktywnie poszukuje innowacyjnych pomysłów.*	1 – 2 – 3 – 4 – 5
OC2	*W naszej firmie omawiamy nowe sposoby prowadzenia działalności.*	1 – 2 – 3 – 4 – 5
OC3	*Wykazywanie inicjatywy często zyskuje uznanie, dlatego pracownicy czują się zachęcani do generowania nowych pomysłów.*	1 – 2 – 3 – 4 – 5
OC4	*Pracownicy, którzy opierają się innowacjom lub postrzegają innowacje jako zbyt ryzykowne, są raczej wyjątkiem niż regułą.*	1 – 2 – 3 – 4 – 5
Zasoby ludzkie (HR)		
HR1	*Oczekujemy dużej innowacyjności i kreatywności od naszych pracowników.*	1 – 2 – 3 – 4 – 5
HR2	*Programy szkoleniowe zwiększające indywidualną innowacyjność pracowników są oferowane regularnie.*	1 – 2 – 3 – 4 – 5
HR3	*Wyniki pracowników w zakresie innowacyjności są systematycznie oceniane.*	1 – 2 – 3 – 4 – 5
HR4	*Nasi pracownicy są zachęcani do aktywnego poszukiwania innowacyjnych pomysłów.*	1 – 2 – 3 – 4 – 5

Kod	Stwierdzenia	Ocena
Orientacja rynkowa (MO)		
MO1	*Nieustannie staramy się odkrywać dodatkowe potrzeby naszych klientów, z których oni sami nie zdają sobie sprawy.*	1 – 2 – 3 – 4 – 5
MO2	*Nowoopracowane usługi naszej firmy oferują unikalne korzyści dla naszych klientów.*	1 – 2 – 3 – 4 – 5
MO3	*Nowoopracowane usługi naszej firmy rozwiązują problemy naszych klientów.*	1 – 2 – 3 – 4 – 5
MO4	*Nowoopracowane usługi naszej firmy zapewniają duże korzyści dla naszych klientów.*	1 – 2 – 3 – 4 – 5
Orientacja technologiczna (TO)		
TO1	*Nowe rozwiązania technologiczne są chętnie akceptowane i wykorzystywane w naszej firmie.*	1 – 2 – 3 – 4 – 5
TO2	*Systematycznie poszukujemy nowych technologii wewnątrz i na zewnątrz branży.*	1 – 2 – 3 – 4 – 5
TO3	*Używamy technologii, które mają wpływ lub powodują istotne zmiany w całej branży.*	1 – 2 – 3 – 4 – 5
TE4	*Systemy informatyczne/technologie informacyjne są źródłem przewagi konkurencyjnej naszej firmy.*	1 – 2 – 3 – 4 – 5

Kod	Stwierdzenia	Ocena
Wyniki działalności (OP)		
P1	*Nasza firma ma szczególną zdolność do wprowadzania nowych, skutecznych usług.*	1 – 2 – 3 – 4 – 5
P2	*Wzrost sprzedaży w naszej firmie można określić jako udany.*	1 – 2 – 3 – 4 – 5
P3	*Zadowolenie klientów z naszej firmy można określić jako udane.*	1 – 2 – 3 – 4 – 5

Informacje o respondentach

Doświadczenie zawodowe:	Stanowisko:
○ poniżej 2 lat<br>○ 2-5 lat<br>○ 6-10 lat<br>○ 11-20 lat<br>○ ponad 20 lat	○ Prezes<br>○ Dyrektor<br>○ Kierownik<br>○ Specjalista

Informacje o firmie respondenta

Obecność na rynku:	Wielkość (liczba zatrudnionych):	Rodzaj świadczonych usług
○ do 2 lat<br>○ 2-5 lat<br>○ 6-10 lat<br>○ 11-20 lat<br>○ ponad 20 lat	○ do 9 pracowników<br>○ 10-49<br>○ 50-249<br>○ powyżej 249	○ Usług informatyczne<br>○ Usługi badań i analiz technicznych<br>○ Usługi inżynieryjne i architektoniczne
